# Effects of Multi-Pass Butt-Upset Cold Welding on Mechanical Performance of Cu-Mg Alloys

**DOI:** 10.3390/ma18245641

**Published:** 2025-12-15

**Authors:** Yuan Yuan, Yong Pang, Zhu Xiao, Shifang Li, Zejun Wang

**Affiliations:** 1 Standards & Metrology Research Institute, China Academy of Railway Sciences Corp., Ltd., Beijing 100081, China; 2 School of Materials Science and Engineering, Central South University, Changsha 410083, China

**Keywords:** Cu-Mg alloy, multi-pass cold welding, bonding mechanism, microstructure evolution, severe plastic deformation (SPD)

## Abstract

Joining high-strength, cold-drawn Cu-Mg alloy conductors is a critical challenge for ensuring the reliability of high-speed railway catenary systems. This study investigates the evolution of mechanical properties and microstructure in Cu-0.43 wt% Mg alloy wires joined by multi-pass butt-upset cold welding without special surface preparation. High-integrity joints were achieved, exhibiting a peak tensile strength of 624 MPa (~96% of the base material’s strength). After four upsetting processes, the tensile strength of the weld can reach 90% of the original strength, and the gains from subsequent upsetting processes are negligible. Microstructural analysis revealed the joining process is governed by localized severe shear deformation, which forges a distinct gradient microstructure. This includes a transition zone of fine, equiaxed-like grains formed by dynamic recrystallization/recovery, and a central zone featuring a nano-laminar structure, high dislocation density, and deformation twins. A multi-stage dynamic bonding mechanism is proposed. It progresses from initial contact via thin film theory to bond consolidation through a “mechanical self-cleaning” process, where extensive radial plastic flow effectively expels surface contaminants. This work clarifies the fundamental bonding principles for pre-strained, high-strength alloys under multi-pass cold welding, providing a scientific basis to optimize this heat-free joining technology for industrial applications.

## 1. Introduction

Copper alloys possessing both high mechanical strength and excellent electrical conductivity represent a class of indispensable materials for demanding industrial applications [1]. Among them, the Cu-Mg alloy has become the preferred material for contact wires of high-speed railway networks with speeds exceeding 300 km/h due to its superior combination of tensile strength, conductivity, wear resistance, and resistance to high-temperature softening at a moderate cost [2,3,4,5,6]. The continuous development of next-generation railway systems necessitates even higher performance from these critical components, driving ongoing research into advanced Cu-based conductor materials [7].

The requisite high strength in commercial Cu-Mg wires is typically achieved through severe plastic deformation (SPD) processes, such as multi-pass cold drawing. Extensive research has validated the efficacy of SPD techniques, including equal channel angular pressing (ECAP) and cryogenic rolling, in significantly enhancing Cu-Mg alloy mechanical performance through mechanisms like grain refinement and increased dislocation density [8,9,10]. However, inherent limitations in manufacturing equipment and logistics often preclude the production of single-piece wires of sufficient length for extensive railway contact lines, as specified by railway engineering standards [11]. Consequently, a robust and reliable joining technology capable of producing high-integrity bonds between segments of high-strength Cu-Mg wire is critically needed to ensure manufacturing continuity, facilitate installation, and maintain the operational integrity of high-speed rail power systems.

Cold welding, a solid-state joining process that achieves metallurgical bonding through substantial plastic deformation at ambient temperatures [12], offers a compelling solution. Its principal advantage lies in the circumvention of heat input, thereby precluding the formation of detrimental microstructural features such as heat-affected zones or brittle intermetallic phases commonly associated with fusion welding techniques [13,14]. Significantly, cold-welded joints, particularly those produced via butt upsetting configurations, can exhibit mechanical integrity approaching or even exceeding that of the parent material, achieving near 100% joint efficiency in various metallic systems [12,15,16,17,18]. This makes the technique highly promising for the specific application of joining high-strength Cu-Mg wires.

Understanding and clarifying the cold-welding joint connection mechanism is crucial for process optimization in industrial practice. Seminal work, such as Bay’s “thin film theory”, provided initial insights into interfacial bonding, emphasizing surface expansion, fracture of brittle surface films, and extrusion of virgin material [19]. Subsequent models have explored the roles of microscale phenomena, including diffusion [20], recrystallization [21], and dislocation dynamics [22]. However, a significant portion of prior mechanistic studies focused on pure metals or alloys in an initially undeformed, annealed state, such as pure aluminum or copper [23,24,25]. These studies often employed relatively minor deformation and stressed the necessity of meticulous surface preparation. These idealized conditions diverge considerably from the practical scenario of joining heavily cold-worked materials, which present unique challenges due to their high flow stress and limited ductility [26]. The complex interplay between severe deformation, microstructural evolution (including potential dynamic recovery or recrystallization phenomena), and the atomic-level bonding process in such pre-strained materials requires further elucidation.

This study, therefore, investigates the effect of multi-pass butt upsetting cold welding on the mechanical properties of cold-drawn, high-strength Cu-0.43 wt% Mg wires without special surface pretreatment. The effect of the number of upsetting passes on the mechanical properties (tensile strength and elongation) and the cross-scale microstructural evolution of the joints was systematically studied. A multi-stage dynamic bonding mechanism is proposed to explain the joining of high-strength Cu-Mg alloys, providing fundamental insights and practical references for the application of this technology in railway conductor connections.

## 2. Materials and Methods

**Figure 1:**
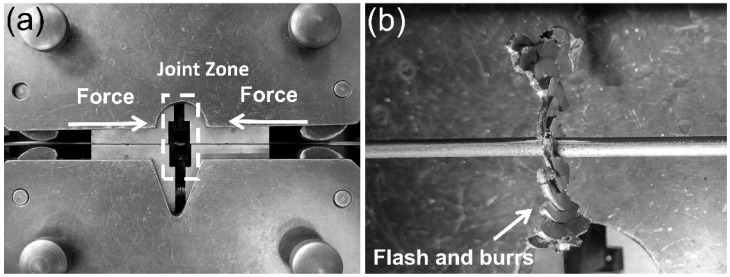
Cold welding of Cu-Mg alloy wires: (**a**) The assembly and setting of the cold-welding machine. (**b**) Typical welded joint after 4 upsetting passes.

The as-received copper alloy wires, composed of Cu-0.43 wt% Mg, were in a cold-drawn state with a diameter of 2.8 mm. The copper–magnesium alloy wires were connected using an AC1510-B hydraulic cold-welding machine provided by Shanghai Shenchen Cable Equipment Co., Ltd. (Shanghai, China). Before welding, the welding surface was mechanically cut to expose a clean, bare surface, and then immediately extruded. The welding process was carried out in an atmospheric environment of 16.1 °C and 45% relative humidity. As shown in Figure 1a, the wire end was clamped in a fixture for upsetting deformation. The final gap between the dies was approximately 1 mm, the approach speed between the fixtures was approximately 2 mm/s, the total strain was 2.6, and the strain rate was 0.65 s^−1^. The macroscopic morphology of the welded joint is shown in Figure 1b. The welded joints’ flash and burrs were removed manually using pliers.

The mechanical properties and microstructure of materials in different states, including samples with varying numbers of upsetting passes, were analyzed. The tensile strength of the wire and the joint was measured using a UH-200KN Shimadzu universal test machine (Shimadzu HandelsgmbH, Korneuburg, Austria) with an initial rate of 2%/min at room temperature. The weld is located at the center of the sample, along the normal direction of the sample placement. The strain measurement method is the crosshead displacement strain measurement method. Three tests were performed for each condition. Tensile specimens were cut along the length of the wire. For welded samples, the joint was placed in the middle of the tensile specimen, and the welded joint and its surroundings were further polished with 800-grit sandpaper before testing to avoid the influence of residual burrs. At least three specimens were tested for each condition. The microstructure of the material was analyzed using various characterization techniques, including optical microscopy (OM, VHX-X1, KEYENCE, Shanghai, China), scanning electron microscopy (SEM, Zeiss EVO MA 10, Oberkochen, Germany), and transmission electron microscopy (TEM, MIRA4 LMH, TESCAN, Brno, Czech Republic). The metallographic samples were mechanically ground and polished, then etched with a reagent containing FeCl_3_ and HCl, and observed using a Leica DM2500M metallographic microscope (Shanghai, China). For EBSD examination, the samples were first mechanically polished, then subjected to ion milling using a Gatan 695 (Pleasanton, CA, USA) precision ion polishing system, and observed using a Zeiss EVO MA 10 scanning electron microscope equipped with an Oxford EBSD detector (Zeiss, Gemany). EBSD step size: 0.15 μm, accelerating voltage: 20 kV. The transmission electron microscopy samples were prepared by mechanical polishing combined with ion milling (Gatan 695, CA, USA) and observed using a Gatan F20 transmission electron microscope operating at 200 kV.

## 3. Results

### 3.1. The Mechanical Properties of Cu-Mg Alloy Before and After Cold Welding

**Figure 2:**
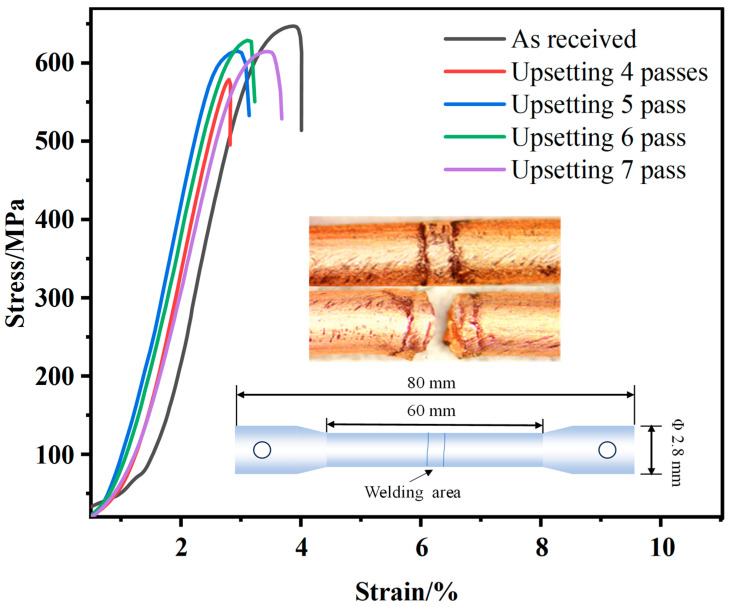
Stress–strain curves of Cu-Mg wire at as-received and after 4–7 passes upsetting process. Inset images show the photo of the original and failure joint by butt-upset cold welding, and the schematic graph of the Cu-Mg wire specimen.

Three copper–magnesium alloy wires from each state were tested for tensile strength, and the results are shown in Table 1, which lists the mechanical properties of Cu-Mg alloy wires before and after cold welding. The ultimate tensile strength of the initial Cu-0.43 wt% Mg alloy wires is 649 MPa, and the corresponding elongation is 4.0%. After four passes of upsetting, the strength and elongation of the welded joint decreased compared to the initial Cu-Mg wires, which were 582 MPa and 2.8%, respectively. Nevertheless, this performance exceeds most other reported welding methods and meets the requirements of contact wires for high-speed railways. The tensile strength of the welded joint initially increases and then decreases as the number of upsetting times increases. After six upsetting times, it reaches a maximum of 624 MPa, but decreases to 613 MPa at the seventh time. The stress–strain curve is shown in Figure 2. It can be seen that the yield strength of the material after cold pressing is 435 MPa, which is slightly lower than the 448 MPa of the matrix. However, the tensile slope of the material after cold pressing is significantly increased. This is mainly due to the high density of dislocations and interface strengthening introduced by cold pressing, which makes the material exhibit stronger resistance to deformation. The corresponding elongation remains at around 3%. The weld joint exhibited necking behavior, demonstrating typical characteristics of ductile fracture. As the number of upsetting passes increased, elongation slightly increased, which is in line with experimental expectations. With the increase in the number of upsetting passes, weld defects decreased, the weld center area widened, and weld fracture became more difficult.

### 3.2. Microstructure of the Welded Joints

**Figure 3:**
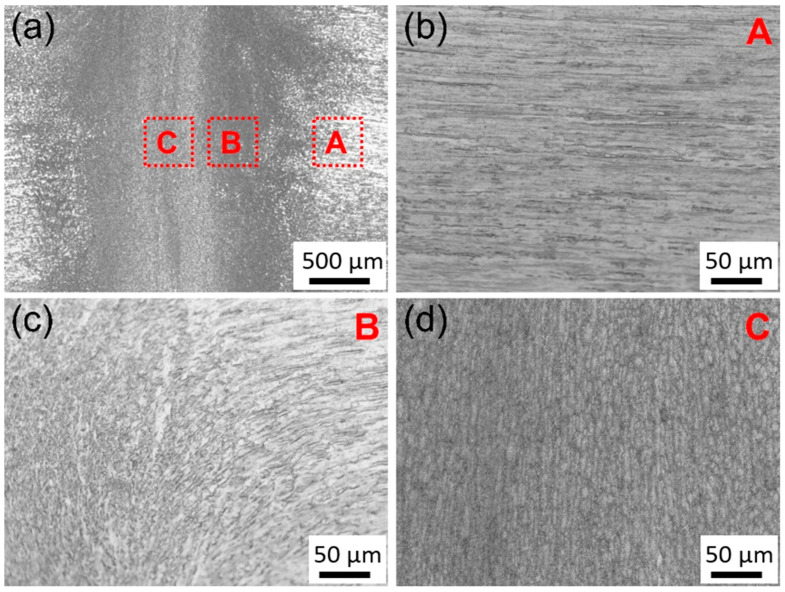
Metallographic structure of the welded joints after 4 passes upsetting: (**a**) Welded joint can be divided into three typical zone, as marked by A, B, and C. (**b**) The base material, corresponding to area A. (**c**) The transitional zone, related to area B. (**d**) The core zone, amplified form area C.

Figure 3 shows the metallographic structure of the welded joint after four upsets. As shown in Figure 3a, the microstructural morphology of the central and peripheral areas of the joint differs significantly. The microstructure characteristics divide the joint into three areas, matrix, transition, and central areas, corresponding to areas A, B, and C in Figure 3a. Figure 3b shows the metallographic structure of the matrix, where elongated grains are evenly distributed along the length direction of the wire, forming a typical fiber-like structure with a width of approximately 5 μm. Figure 3c is the metallographic diagram of the transition zone between the matrix and the joint core, where the direction of metal flow changes smoothly from parallel to perpendicular to the length of the wire, indicating that shear deformation occurs locally in the material. The width of the transition area is about 100 μm, and the whole structure is dense. Few defects, such as cracks and adiabatic shear bands, are observed. Figure 3d gives the metallographic structure of the center of the joint, where the grain arrangement is perpendicular to that in the matrix.

**Figure 4:**
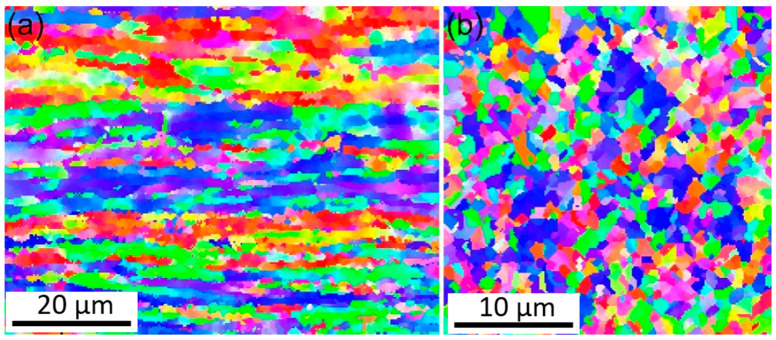
IPF mapping of (**a**) the base material and (**b**) the transition zone in the welded joint.

Figure 4 shows the inverse pole figure (IPF) mapping of the matrix of Cu-Mg wires and the transition zone of the welded joint measured by EBSD. As shown in Figure 4a, the initial structure of Cu-Mg wires is mainly composed of fibrous shape elongated grains, which is consistent with the results observed in metallography (Figure 3b). Some grains within the fibrous shape structure exhibit a graduated change in orientation color, indicating the presence of misorientations and sub-grain microstructure. The transition region exhibits mainly fine equiaxed-like grains, whose arrangement is weakly related to the flow direction of the metal, as shown in Figure 4b. The internal orientation colors of these equiaxed-like grains tend to be consistent, similar to a typical recrystallized structure, indicating the occurrence of grain refinement and reorientation during the upsetting process.

**Figure 5:**
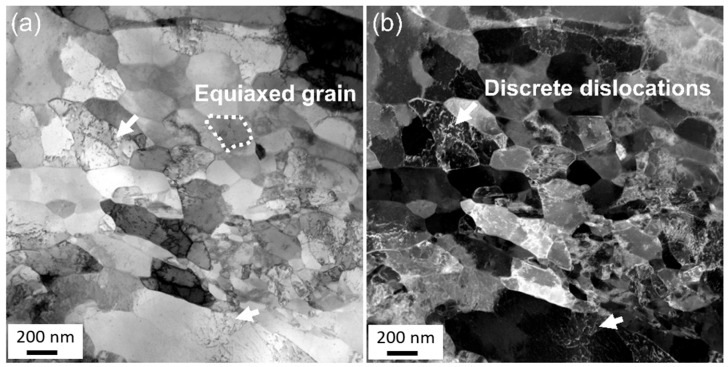
TEM images of the transition zone in the joint: characterized by equiaxed-like grains. (**a**) Bright field image. (**b**) Dark field image. Typical outline of grains and defects such as dislocations are labeled by white arrows.

Figure 5 illustrates the microstructure of the transition zone in the welding joint observed by TEM. The grains in the transition region are small, with a diameter of about 200 nm, some even smaller than 100 nm, and most of them have an equiaxed shape. No second phase is observed inside the grains, which is because according to the Cu-Mg binary phase diagram, during high-temperature heat treatment, magnesium completely dissolved in the Cu matrix and could not form a precipitated phase (Figure S1). In some grains, discrete dislocations are observed instead of dislocation cells or cell blocks. This phenomenon is more obvious in the dark field image, as marked by the white arrows in Figure 5.

**Figure 6:**
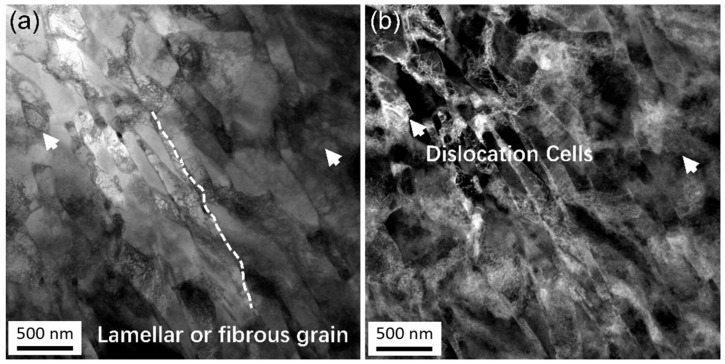
TEM images near the core zone of the joint: characterized by lamellar or fibrous shape grains. (**a**) Bright field image. (**b**) Dark field image. Typical profile of grains and defects, such as dislocation cells, are labeled by white arrows.

**Figure 7:**
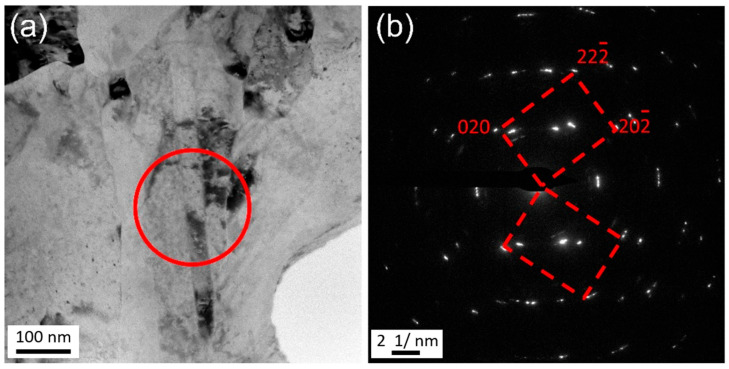
TEM Images of the central zone in the joint: formation of nano-sized twins. (**a**) Bright field image. (**b**) Corresponding selected area electron diffraction patterns.

Figure 6 illustrates the microstructure near the center of the joint observed using TEM. The crystal grains in the center of the joint exhibit a lamellar or fibrous shape morphology, with a width of approximately 100 nm. This microstructure, analogous to that of the initial Cu-Mg wire, reflects the direction of metal flow. In addition to the grain morphology, two significant differences can be observed in comparison to the microstructure of the transition zone. Firstly, the dislocation density inside the grain is significantly higher than that in the transition zone. Secondly, the dislocation morphology within the grain is identified as dislocation cells or cell blocks, as marked by the white arrows in Figure 6b, instead of the discrete dislocations presented in the transition zone (Figure 5). In addition to elongated nanocrystals with a high density of dislocations and sub-grain microstructure, nanotwin was also observed in some regions of the central area of the weld head as shown in Figure 7. These results show that the metal flow of Cu-Mg alloy undergoes severe plastic deformation after passing through the transition zone.

**Figure 8:**
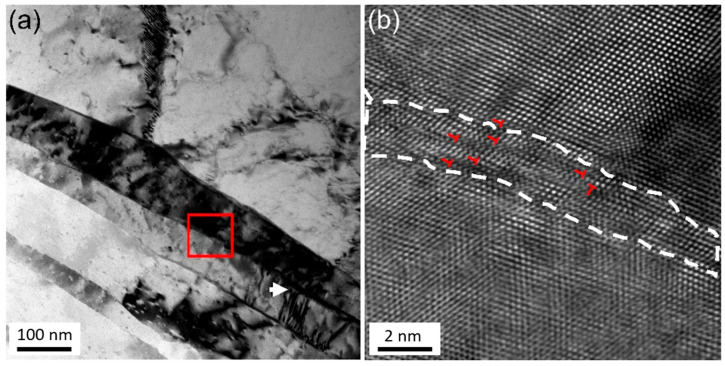
(**a**) Bright field image of two adjacent grains in the core zone of the welded joint. (**b**) HR-TEM of the grain boundaries, corresponding to the red-marked area. The interfacial structure and some dislocations within the boundary are labeled.

Figure 8 shows the microstructure of the bonding interface of the welded joint. The grains are elongated and aligned perpendicular to the length of the Cu-Mg alloy wire. The interface between adjacent grains is relatively straight, but not a straight-line interface like twins. The dislocation density inside the grain is not high, and discrete dislocations can be observed at the interface of adjacent grains, as marked by the white arrow in Figure 8a. Figure 8b shows the HR-TEM image corresponding to the interface area marked by the box in Figure 8a. The atomic arrangement of adjacent grains becomes chaotic at the interface. The grains on both sides are approximately in the <110> direction. Still, there is a misorientation between them, which cannot be identified as a coincidence site lattice (CSL) crystal boundary [27]. The thickness of the interface is about 2 nm, and some edge dislocations can be indexed through inverse fast Fourier transform (FFT) analysis [28]. These dislocations would easily move from one side of the interface to the other during the upsetting process of cold welding.

It is worth noting that the grain boundary depicted in Figure 8 cannot be definitively concluded as the exact interface where the Cu-Mg alloy wires on both sides are bonded. The grain boundary shown in Figure 8 is representative of the core area of the welding joint. It is believed that the real bonding interface is indistinguishable from the interface observed in Figure 8.

### 3.3. Effect of Upsetting Pass on Microstructure

**Figure 9:**
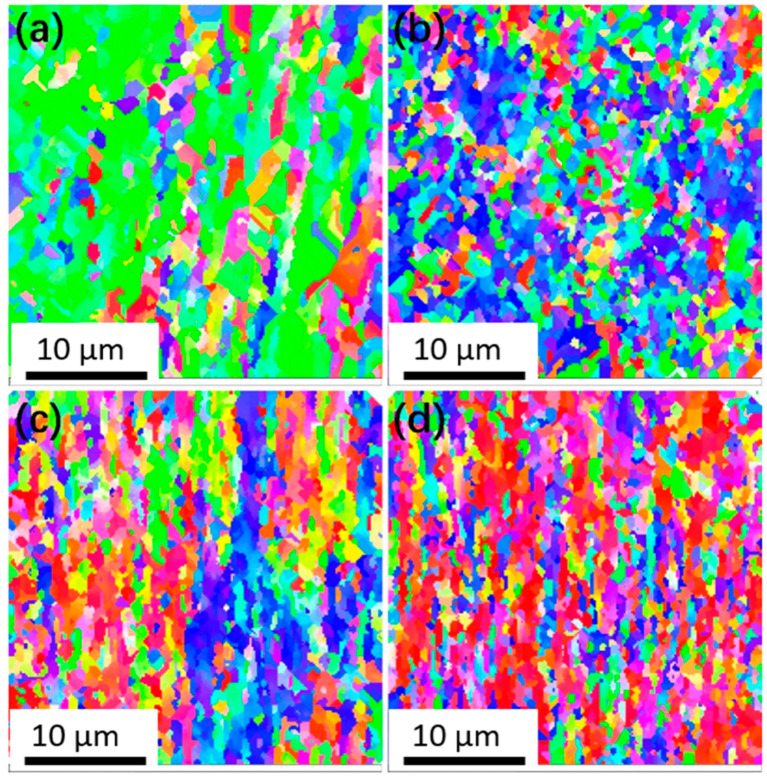
EBSD mapping (IPF) of the central zones in the welded joints with various upsetting passes: (**a**) upsetting 4 passes; (**b**) upsetting 5 passes; (**c**) upsetting 6 passes; (**d**) upsetting 7 passes.

Figure 9 shows the IPF mapping of the central area of the welded joints under different upsetting passes measured by EBSD. After four passes and above of upsetting, the central area of the welded joint exhibits fine fiber-like grains and a heavily deformed structure. Some grains show graduated orientation colors, indicating a rich sub-grain structure. This result is consistent with the results observed by transmission electron microscopy in Figure 6. However, the crystal grains measured by EBSD in Figure 9 are notably larger than the crystal grains observed by TEM in Figure 6, which may be due to the lower resolution of the EBSD technology used.

**Figure 10:**
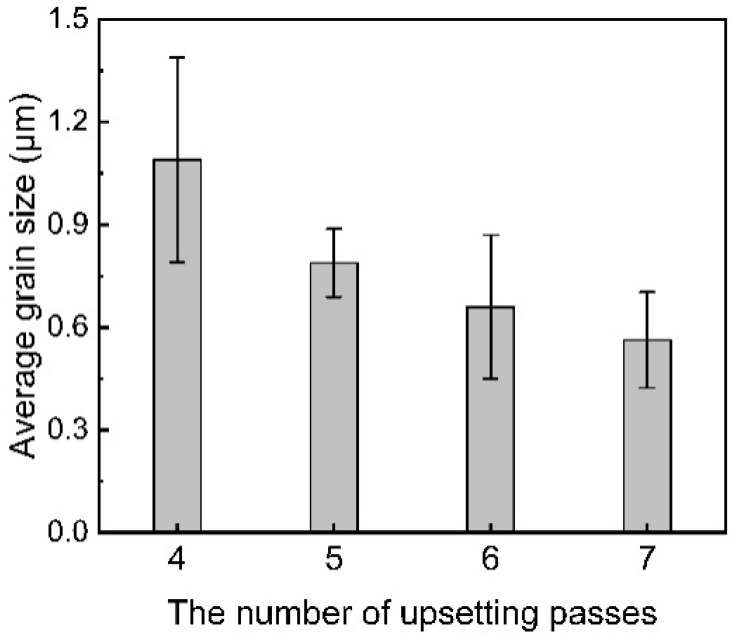
The average grain size of the central zones in the welded joints with various upsetting passes.

Figure 10 displays the average grain size of the central area of the welding joint obtained through different upsetting passes based on EBSD results. Overall, the grain size of the central areas in the welded joints slightly decreases as the number of upsetting times increases. After four upsetting times, the central grain size of the joint is 1.09 μm, while after seven upsetting times, the average grain size drops to 0.56 μm, reaching the ultra-fine grain level. The microstructure of the weld center region is non-uniform, and the gain is affected by the selected measurement area and the number of samples: EBSD scans a larger area, yields more grain samples that meet the HAGB conditions, and has high statistical reliability. TEM, due to limitations in sample preparation and field of view, can usually only analyze a very small area. We acknowledge that its statistical performance is far lower than EBSD, but it accurately reveals the nanoscale structural features of this local region.

**Figure 11:**
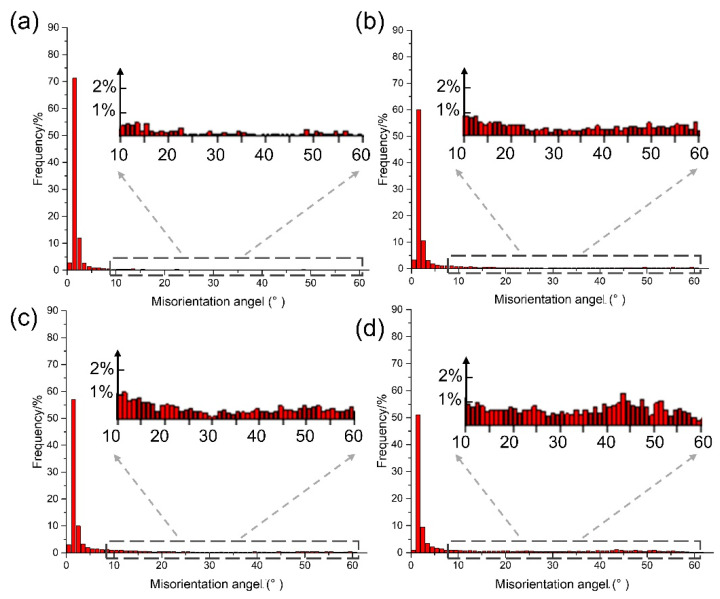
Misorientation distribution of the central zones in the welded joints with various upsetting passes: (**a**) upsetting 4 passes; (**b**) upsetting 5 passes; (**c**) upsetting 6 passes; (**d**) upsetting 7 passes.

Figure 11 illustrates the misorientation distribution of grain boundaries (GB) in the center of the joint under different upsetting times. When upsetting is performed four times, small-angle GB with angles ≤ 5° dominate, sub-grains with angles < 2° account for 83%, and small-angle GB account for 10% of grains with angles of 2~3°. As the number of upsetting increases, the small-angle GB gradually transform into high-angle GB. After upsetting seven times, the sub-grains with angles < 2° were reduced to 52%, the small-angle GB with 2~3° accounted for 13%, and the large-angle GB with angles > 15° increased to 24%.

## 4. Discussion

This paper investigates the effects of upsetting cycles on the mechanical strength and microstructure of cold-welded joints. High-strength cold-drawn copper–magnesium alloy wires that have undergone seven upsetting cycles achieve a joint tensile strength of up to 96% of the base material. This section will discuss in detail the evolution of the microstructure, the dynamic bonding mechanism under intense plastic deformation, and the relationship between microstructure and mechanical properties.

### 4.1. Deformation Inhomogeneity and the Evolution of a Gradient Microstructure

The plastic deformation during cold welding is highly localized within the joint region, resulting in the formation of a distinct gradient microstructure. As evidenced by the macroscopic morphology of the joint (Figure 1b), material predominantly flows radially into the flash rather than being axially compressed, indicating that shear deformation is the governing mode [29]. This intense shear flow leads to the development of three characteristic zones extending from the base material to the joint centerline. The base material zone (Area A) retains the fibrous shape, work-hardened microstructure from the initial cold drawing process. In contrast, the transition zone (Area B) undergoes a fundamental microstructural transformation. The original fibrous shape grains are replaced by fine, equiaxed-liked grains with a relatively low internal dislocation density (Figure 4b and Figure 5). Finite element simulation analysis shows that when the effective strain reaches 2.5 or higher, the recrystallization temperature of the material decreases significantly due to the large deformation [30,31]. Simultaneously, local temperature rise occurs during large plastic deformation, creating favorable conditions for recrystallization nucleation. Because heat transfer to the sample and mold is rapid during cold pressure welding, temperature changes cannot be accurately measured. However, at the weld joint, the local temperature may reach the temperature threshold for dynamic recrystallization, and the dynamic recrystallization process may occur. This conjecture is consistent with the conclusions in the cited references [30,31]. TEM analysis reveals that CuMg alloys undergo dynamic recrystallization (DRX) or significant dynamic recovery (DRV) under the intense plastic deformation conditions of cold-press welding [32,33], which acts as a softening and structural reorganization mechanism to accommodate the extreme localized strain, consuming stored strain energy to form new, strain-free grains. Subsequently, as the material flows from the transition zone to the interface, it is subjected to the most severe compression and extrusion in the central zone (Area C), undergoing typical severe plastic deformation (SPD) [34]. This results in further grain refinement and elongation, forming a nano-laminar or fibrous shape structure oriented perpendicular to the wire axis (Figure 6). Concurrently, a high density of dislocations accumulates and arranges into dislocation cells and sub-grain boundaries, with nano-twinning being activated in localized areas [35] (Figure 7). These features are characteristic of significant work hardening during SPD. Consequently, the entire joint can be regarded as a gradient material, forged by the interplay of shear, dynamic recovery/recrystallization, and severe plastic deformation.

### 4.2. The Dynamic Bonding Mechanism in Multi-Pass Cold Welding

**Figure 12:**
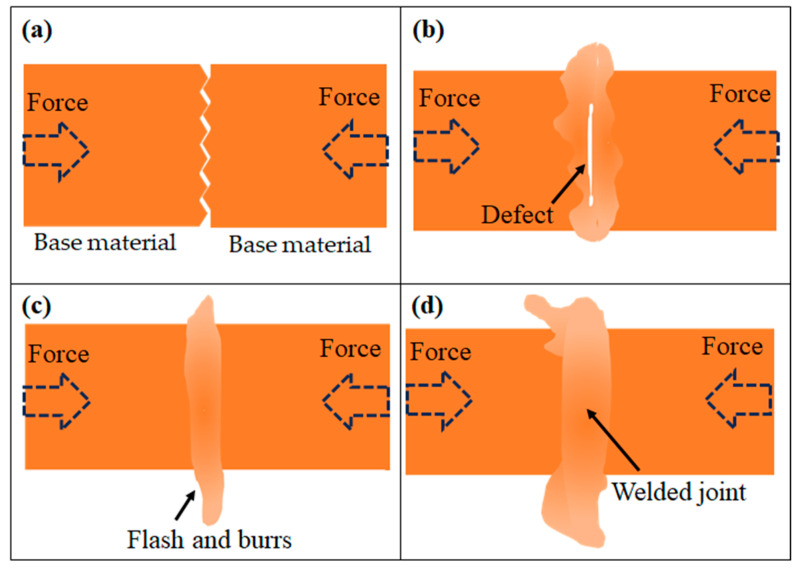
Schematic diagram of multi-pass cold welding: (**a**) As received, (**b**) initial stage, (**c**) second stage, (**d**) final stage.

The experimental finding that a minimum of four upsetting passes is required to achieve a robust joint underscores that interfacial bonding is a progressive and dynamic process. A three-stage model is proposed to elucidate this mechanism, as shown in Figure 12. The initial stage, encompassing the first one to two passes, aligns with the classic thin film theory [19]. Under immense pressure, brittle surface layers such as oxides and contaminants on the wire ends fracture, allowing the extrusion of nascent, clean metal through the cracks to form localized, discontinuous metallic bonds. For a high-strength, work-hardened material, however, these initial bonds are insufficient to create a strong joint, as large unbonded areas and trapped contaminants remain.

The second stage, occurring between the second and fourth passes, is critical for achieving a sound bond. Continued upsetting drives severe radial plastic flow at the interface, which functions as a highly effective “mechanical self-cleaning” process. This flow extrudes fragmented oxide particles and other impurities from the central interface region outwards into the flash. As the interface is progressively purified, the contact area of nascent metal surfaces increases substantially, leading to the rapid expansion and consolidation of a large-scale metallurgical bond.

The final stage is characterized by tensile strength and microstructural morphology, which occurs after approximately four passes. By this point, the interface has been effectively cleaned and a strong metallurgical bond has formed, capable of facilitating coordinated deformation across the joint. Subsequent deformation cycles therefore act on an integrated joint, causing the microstructural evolution to enter a quasi-steady state [36]. Further passes only induce marginal grain refinement (Figure 10) and a modest increase in the fraction of high-angle grain boundaries (Figure 11), without significantly altering the overall structure (Figures S2–S5). At the atomic scale, the accommodation of lattice mismatch across the newly formed interface is mediated by dislocation motion, such as cross-slip and climb [37], ultimately creating a stable interface that is indistinguishable from a conventional high-angle grain boundary (Figure 8).

### 4.3. Microstructure and Mechanical Property

Once the cold-pressed welded joint successfully establishes an effective connection, its conductivity exhibits a high degree of consistency. The conductivity indicators are very similar to those of the base material, and all can reach more than 99% of the conductivity of the base material [38]. The changes in the mechanical properties of the joint are closely related to its microstructure. The mechanical performance of the joint is a macroscopic reflection of its gradient microstructure. The ultimate tensile strength (approx. 624 MPa), slightly lower than that of the base material (649 MPa), is determined by a competition between strengthening and softening mechanisms. The central zone is strengthened by the formation of ultra-fine and nano-scale grains via SPD, which provides a significant Hall–Petch strengthening contribution [39]. Conversely, the occurrence of DRX/DRV in the transition zone is a classic softening process that partially counteracts the work hardening. The final strength of the joint represents a volume-weighted average of these competing effects across the load-bearing cross-section. Figure S6 demonstrated the hardness gradient of transverse microhardness profiles (base → joint → base). After four rounds of upsetting, the tensile strength of the Cu-Mg wire weld zone can be restored to 90% of that of the base material, which can meet the requirements of practical applications. The recovery of this performance is mainly due to two factors: first, the defects at the weld interface are significantly reduced; second, local melting and recrystallization occurred at the interface of the two Cu-Mg wires, forming a homogeneous central region of metallurgical bonding. In the subsequent strain process, the strength improvement brought about by work hardening and the softening effect caused by dynamic recovery reach a dynamic balance, so that the mechanical properties of the material no longer change significantly (Figure S7) [40].

### 4.4. Limitations and Future Outlook

While this work elucidates key mechanisms in the cold welding of Cu-Mg alloys, certain limitations exist. The analysis is based entirely on post-mortem characterization, which precludes in situ observation of the dynamic bonding process. Future work could therefore focus on two primary areas. First, Finite Element Method (FEM) simulations could be employed to model the stress–strain fields during the multi-pass upsetting process, enabling a more precise prediction of microstructural evolution in different zones [41]. Second, to support the engineering application of this technology for high-speed railway catenary systems. Future research plans include conducting cold-press welding tests on large-diameter copper rods, fabricating contact wires for fatigue testing under actual working tension and tension fluctuation ranges, and carrying out pantograph–catenary wear tests under current collection conditions. A comprehensive evaluation of the joint’s fatigue performance, wear resistance, and long-term thermal stability is essential [42].

## 5. Conclusions

The following conclusions can be drawn from this investigation:

(1) Multi-pass upsetting cold welding of Cu-Mg alloy wire can achieve tensile stress of up to 624 MPa and 3.5% elongation at break.
(2) During the cold-welding upsetting process, the Cu-Mg contact interface undergoes shear and extrusion deformation, which eventually leads to severe deformation in the central area.
(3) During the multiple upsetting processes, the microscopic control theory of Cu-Mg alloy wire connection is transformed from thin film theory to plastic deformation of dislocation cross-slip.

## Figures and Tables

**Table 1:** Mechanical properties of Cu-Mg alloy before and after welding.

Condition	Tensile Strength/MPa	Yield Strength/MPa	Elongation at Break/%
As received	649 ± 3	448 ± 2	4.0 ± 0.2
Upsetting 4 passes	582 ± 7	412 ± 3	2.8 ± 0.5
Upsetting 5 passes	612 ± 4	431 ± 5	3.1 ± 0.2
Upsetting 6 passes	624 ± 5	435 ± 4	3.2 ± 0.3
Upsetting 7 passes	613 ± 5	427 ± 2	3.5 ± 0.5

## Data Availability

The original contributions presented in this study are included in the article/Supplementary Materials. Further inquiries can be directed to the corresponding authors.

## Supplementary Materials

The following supporting information can be downloaded at https://www.mdpi.com/article/10.3390/ma18245641/s1; Figure S1: The Cu-Mg binary metallographic diagram demonstrates that in Cu alloys with low Mg content, Mg is completely dissolved in the Cu matrix at high temperatures; Figure S2: (a) Inverse pole figure (IPF), (b) Texture distribution map, (c) KAM maps, (d) Copper base grain size map, (e) HAGB/LAGB fractions, (f) Local orientation angles for as received Cu-Mg wires after 4 upsetting processes; Figure S3: (a) Inverse pole figure (IPF), (b) Texture distribution map, (c) KAM maps, (d) Copper base grain size map, (e) HAGB/LAGB fractions, (f) Local orientation angles for welded Cu-Mg wires after 5 upsetting processes; Figure S4: (a) Inverse pole figure (IPF), (b) Texture distribution map, (c) KAM maps, (d) Copper base grain size map, (e) HAGB/LAGB fractions, (f) Local orientation angles for welded Cu-Mg wires after 6 upsetting processes; Figure S5: (a) Inverse pole figure (IPF), (b) Texture distribution map, (c) KAM maps, (d) Copper base grain size map, (e) HAGB/LAGB fractions, (f) Local orientation angles for welded Cu-Mg wires after 7 upsetting processes; Figure S6: Microhardness maps across the weld cross-section of Cu-Mg wire; Figure S7: (a–g) Optical micrographs of Cu-Mg wire joints that have undergone 1–7 upsetting cold-welding processes, respectively.

## References

[1] Yang K., Wang Y., Guo M., Wang H., Mo Y., Dong X., Lou H. (2023). Recent Development of Advanced Precipitation-Strengthened Cu Alloys with High Strength and Conductivity: A Review. Prog. Mater. Sci..

[2] Wu G., Gao G., Wei W., Yang Z., Wu G., Gao G., Wei W., Yang Z. (2019). Electric Contact Material of Pantograph and Catenary. The Electrical Contact of the Pantograph-Catenary System: Theory and Application.

[3] Li Y., Xiao Z., Li Z., Zhou Z., Yang Z., Lei Q. (2017). Microstructure and Properties of a Novel Cu-Mg-Ca Alloy with High Strength and High Electrical Conductivity. J. Alloys Compd..

[4] Zhen G., Kim Y., Haochuang L., Koo J.-M., Seok C.-S., Lee K., Kwon S.-Y. (2014). Bending Fatigue Life Evaluation of Cu-Mg Alloy Contact Wire. Int. J. Precis. Eng. Manuf..

[5] Ma A., Zhu C., Chen J., Jiang J., Song D., Ni S., He Q. (2014). Grain Refinement and High-Performance of Equal-Channel Angular Pressed Cu-Mg Alloy for Electrical Contact Wire. Metals.

[6] Liu Q., Zhang X., Ge Y., Wang J., Cui J.-Z. (2006). Effect of Processing and Heat Treatment on Behavior of Cu-Cr-Zr Alloys to Railway Contact Wire. Metall. Mater. Trans. A.

[7] Wu G., Dong K., Xu Z., Xiao S., Wei W., Chen H., Li J., Huang Z., Li J., Gao G. (2022). Pantograph–Catenary Electrical Contact System of High-Speed Railways: Recent Progress, Challenges, and Outlooks. Railw. Eng. Sci..

[8] Ma M., Li Z., Qiu W., Xiao Z., Zhao Z., Jiang Y. (2019). Microstructure and Properties of Cu–Mg-Ca Alloy Processed by Equal Channel Angular Pressing. J. Alloys Compd..

[9] Zhu C., Ma A., Jiang J., Li X., Song D., Yang D., Yuan Y., Chen J. (2014). Effect of ECAP Combined Cold Working on Mechanical Properties and Electrical Conductivity of Conform-Produced Cu–Mg Alloys. J. Alloys Compd..

[10] Zhang D.T., Li L.I., Zheng Y.F. (2019). High Strength and High Electrical Conductivity CuMg Alloy Prepared by Cryorolling. Trans. Nonferrous Met. Soc. China.

[11] Bai Y., Zhang J., Liu W., Liu X. (2013). Study on Influence of Contact Wire Design Parameters on Contact Characteristics of Pantograph-Catenary.

[12] Bay N. (2011). Cold Welding. Welding Fundamentals and Processes.

[13] Periyasamy P.S., Sivalingam P., Vellingiri V.P., Maruthachalam S., Balakrishnapillai V. (2024). A Review of Traditional and Modern Welding Techniques for Copper. Weld. Int..

[14] Auwal S., Ramesh S., Yusof F., Manladan S.M. (2018). A Review on Laser Beam Welding of Copper Alloys. Int. J. Adv. Manuf. Technol..

[15] Pang Y., Yu H., Xia C., Ni C., Chen C., Wang S., Jia Y., Xiao Z., Yi J. (2025). Microstructural Evolution and Bonding Mechanisms during Multi-Pass Cold Welding of High-Strength Precipitation-Hardened Alloys. J. Mater. Process. Technol..

[16] Tusek J., Markelj F., Jez B. (2003). Influence of Type of Welded Joint on Welding Efficiency. Sci. Technol. Weld. Join..

[17] Wright P., Snow D., Tay C. (1978). Interfacial Conditions and Bond Strength in Cold Pressure Welding by Rolling. Met. Technol..

[18] Peter N.J., Gerlitzky C., Altin A., Wohletz S., Krieger W., Tran T.H., Liebscher C.H., Scheu C., Dehm G., Groche P. (2019). Atomic Level Bonding Mechanism in Steel/Aluminum Joints Produced by Cold Pressure Welding. Materialia.

[19] Bay N. (1979). Cold Pressure Welding—The Mechanisms Governing Bonding. J. Eng. Ind.

[20] Yuntao L., Zeyu D., Jiang Y. (2003). Study on Interfacial Bonding State of Ag-Cu in Cold Pressure Welding. Trans. Tianjin Univ..

[21] Ebbert C., Schmidt H., Rodman D., Nürnberger F., Homberg W., Maier H., Grundmeier G. (2014). Joining with Electrochemical Support (ECUF): Cold Pressure Welding of Copper. J. Mater. Process. Technol..

[22] Latypov R.A., Bulychev V.V., Latypova G.R., Paramonov S.S. (2021). Dislocation Model of the Formation of a Welded Joint in Cold Welding. Mater. Today Proc..

[23] Yücel G., Pinar A.M., Onar V., Özen F., Işitan A. (2025). Effect of the Process Parameters on Joint Performance of Cold Pressure Butt Welded T2 Copper Joints. Proc. Inst. Mech. Eng. Part E J. Process Mech. Eng..

[24] Tabata T., Masaki S., Azekura K. (1989). Bond Criterion in Cold Pressure Welding of Aluminium. Mater. Sci. Technol..

[25] Sahin M., Misirli C. (2012). Properties of Cold Pressure Welded Aluminium and Copper Sheets. Adv. Mater. Res..

[26] McQueen H. (2012). Pressure Welding, Solid State: Role of Hot Deformation. Can. Metall. Q..

[27] Schuh C.A., Kumar M., King W.E. (2005). Universal Features of Grain Boundary Networks in FCC Materials. J. Mater. Sci..

[28] Xia C., Pang Y., Jia Y., Ni C., Sheng X., Wang S., Jiang X., Zhou Z. (2022). Orientation Relationships between Precipitates and Matrix and Their Crystallographic Transformation in a Cu–Cr–Zr Alloy. Mater. Sci. Eng. A.

[29] Kim H.S., Seo M.H., Hong S.I. (2001). Plastic Deformation Analysis of Metals during Equal Channel Angular Pressing. J. Mater. Process. Technol..

[30] Huang Y., Yan X., Ran X.L. (2020). Effect of Compression/Diameter Ratio on Pure Copperbutt Cold-welded Welded Joints. J. Lanzhou Univ. Technol..

[31] Lilleby A., Grong O., Hemmer H. (2010). Cold pressure welding of severely plastically deformed aluminium by divergent extrusion. Mater. Sci. Eng. A.

[32] Takayama A., Yang X., Miura H., Sakai T. (2008). Continuous Static Recrystallization in Ultrafine-Grained Copper Processed by Multi-Directional Forging. Mater. Sci. Eng. A.

[33] Wang Y., Jiao T., Ma E. (2003). Dynamic Processes for Nanostructure Development in Cu after Severe Cryogenic Rolling Deformation. Mater. Trans..

[34] Valiev R.Z., Islamgaliev R.K., Alexandrov I.V. (2000). Bulk Nanostructured Materials from Severe Plastic Deformation. Prog. Mater. Sci..

[35] Dao M., Lu L., Shen Y., Suresh S. (2006). Strength, Strain-Rate Sensitivity and Ductility of Copper with Nanoscale Twins. Acta Mater..

[36] Divinski S.V., Padmanabhan K., Wilde G. (2011). Microstructure Evolution during Severe Plastic Deformation. Philos. Mag..

[37] Schoeck G. (2010). Interaction of Lomer–Cottrell Locks with Screw Dislocations. Philos. Mag..

[38] Huang Y., Yan X., Ran X. (2020). Effect of Compression on Microstructure and Properties of Single Crystal Copper Cold Pressure Welding Joints. Mater. Rep..

[39] Cordero Z.C., Knight B.E., Schuh C.A. (2016). Six Decades of the Hall–Petch Effect–a Survey of Grain-Size Strengthening Studies on Pure Metals. Int. Mater. Rev..

[40] Mecking H. (1981). Strain Hardening and Dynamic Recovery. Dislocation Modelling of Physical Systems.

[41] Campilho R.D. (2023). Advances in the Experimentation and Numerical Modeling of Material Joining Processes. Materials.

[42] Kim Y., Lee K., Cho Y.-H., Guo Z., Koo J.-M., Seok C.-S. (2016). Fatigue Safety Evaluation of Newly Developed Contact Wire for Eco-Friendly High Speed Electric Railway System Considering Wear. Int. J. Precis. Eng. Manuf.-Green Technol..

