# Supplementary material for: Effects of Multi-Pass Butt-Upset Cold Welding on Mechanical Performance of Cu-Mg Alloys
Source: Materials (Basel). 2025 Dec 15;18(24):5641. doi: 10.3390/ma18245641 (PMC12734783; doi:10.3390/ma18245641)
Supplement: Supplementary file 1 [file materials-18-05641-s001.zip › materials-3956652-supplementary.pdf]

Supplementary Materials

Effects of Multi-Pass Butt-Upset Cold Welding on Mechanical Performance of Cu-Mg Alloys

Yuan Yuan [1], Yong Pang [2,*], Zhu Xiao [2], Shifang Li [2] and Zejun Wang [2,*]

[1] Standards & Metrology Research Institute, China Academy of Railway Sciences Corp., Ltd., Beijing 100081, China
[2] School of Materials Science and Engineering, Central South University, Changsha 410083, China
* Correspondence: thgink@126.com (Y.P.); wangzejunmse@163.com (Z.W.)

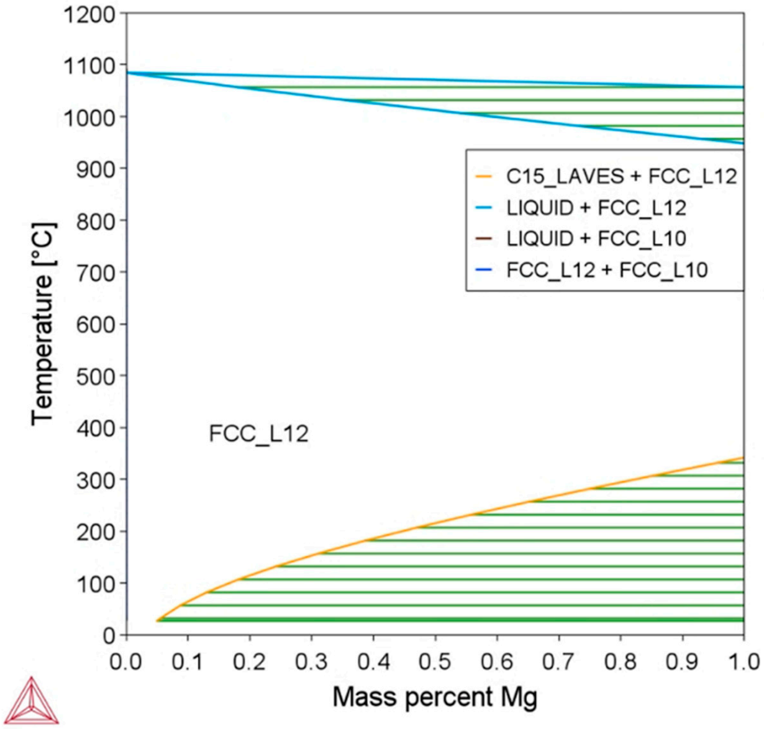

Figure S1. The Cu-Mg binary metallographic diagram demonstrates that in Cu alloys with low Mg content, Mg is completely dissolved in the Cu matrix at high temperatures.

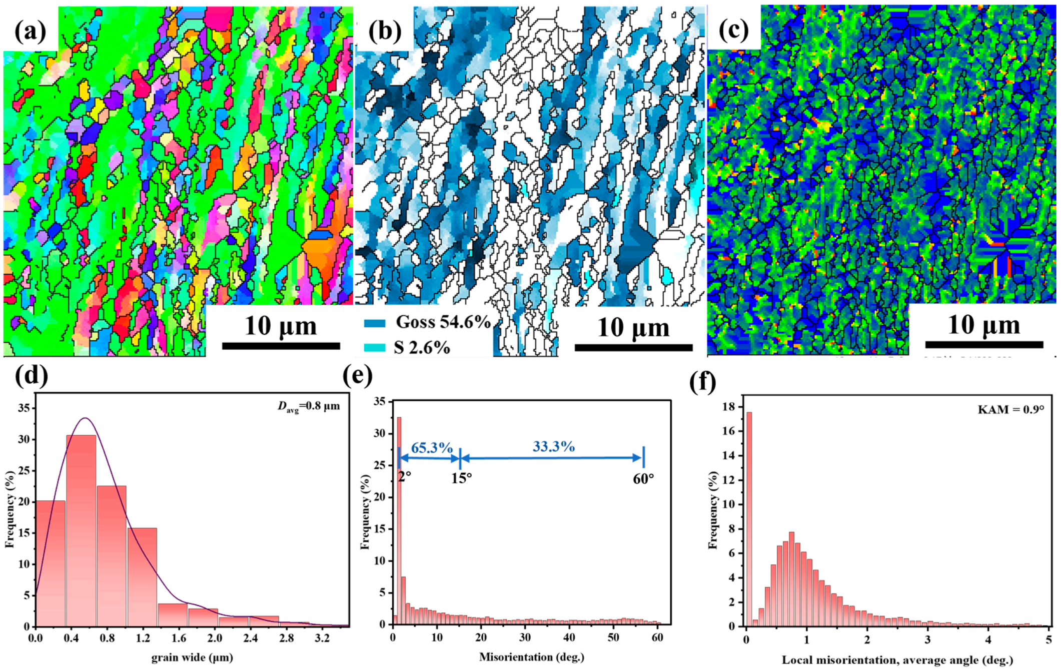

Figure S2. (**a**) Inverse pole figure (IPF), (**b**) Texture distribution map, (**c**) KAM maps, (**d**) Copper base grain size map, (**e**) HAGB/LAGB fractions, (**f**) Local orientation angles for welded Cu-Mg wires after 4 upsetting processes.

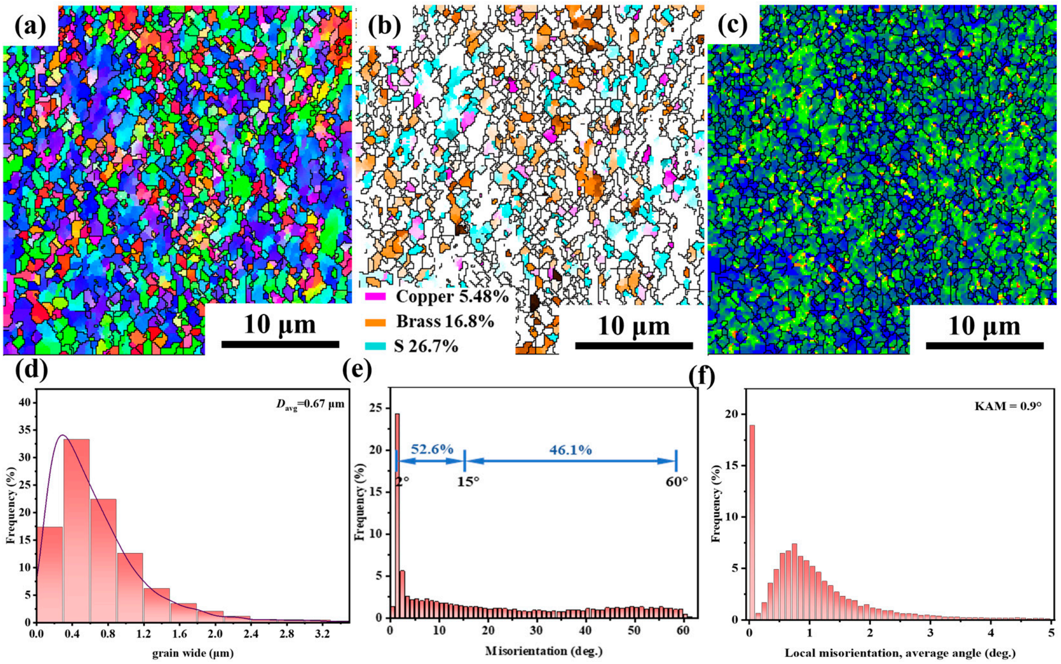

Figure S3. (**a**) Inverse pole figure (IPF), (**b**) Texture distribution map, (**c**) KAM maps, (**d**) Copper base grain size map, (**e**) HAGB/LAGB fractions, (**f**) Local orientation angles for welded Cu-Mg wires after 5 upsetting processes.

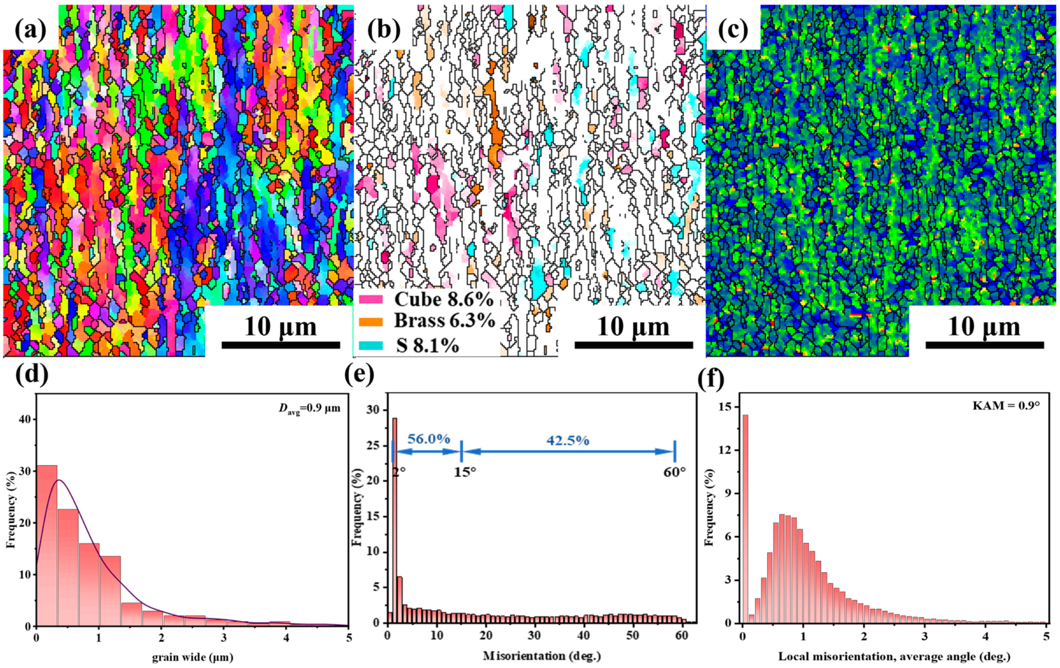

Figure S4. (**a**) Inverse pole figure (IPF), (**b**) Texture distribution map, (**c**) KAM maps, (**d**) Copper base grain size map, (**e**) HAGB/LAGB fractions, (**f**) Local orientation angles for welded Cu-Mg wires after 6 upsetting processes.

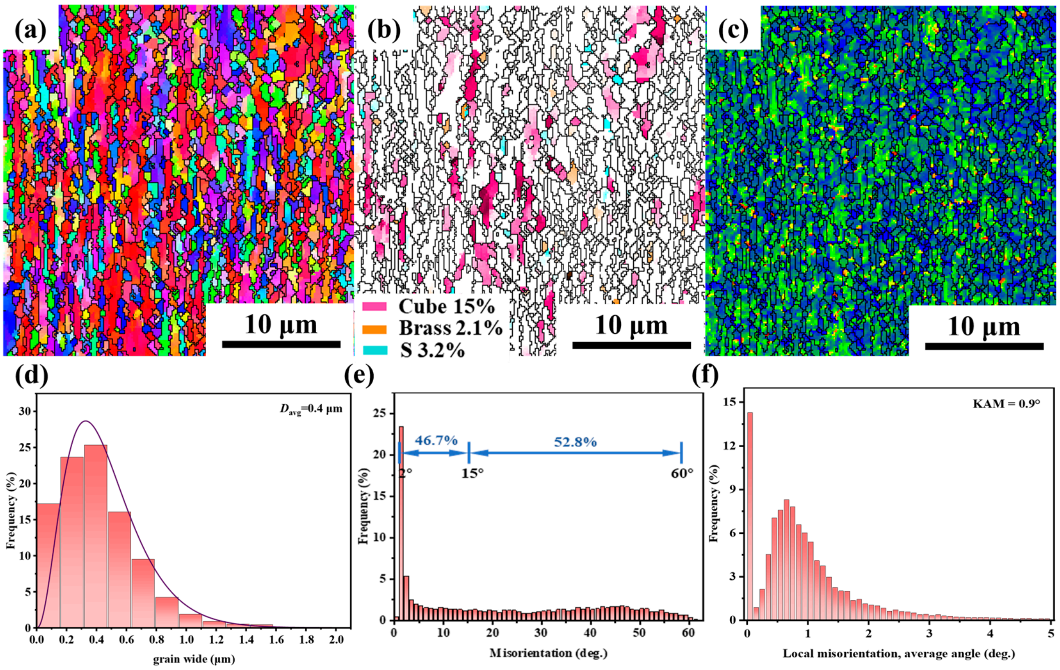

Figure S5. (**a**) Inverse pole figure (IPF), (**b**) Texture distribution map, (**c**) KAM maps, (**d**) Copper base grain size map, (**e**) HAGB/LAGB fractions, (**f**) Local orientation angles for welded Cu-Mg wires after 7 upsetting processes.

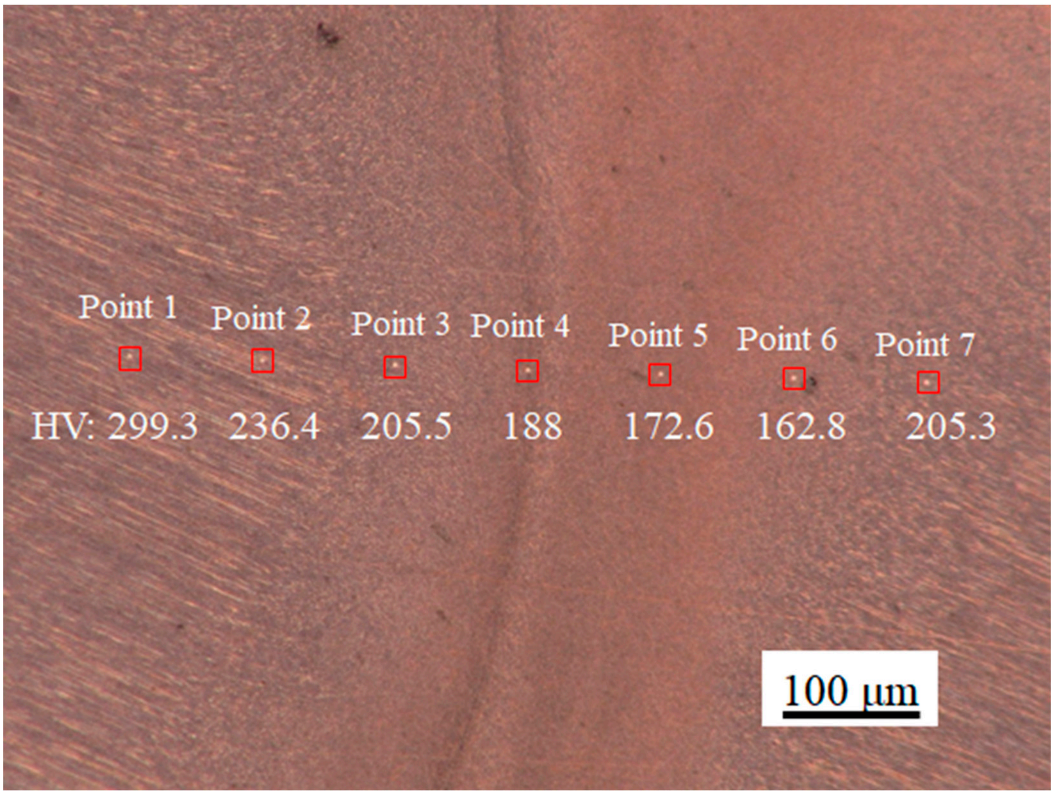

Figure S6. Microhardness maps across the weld cross-section of Cu–Mg wire.

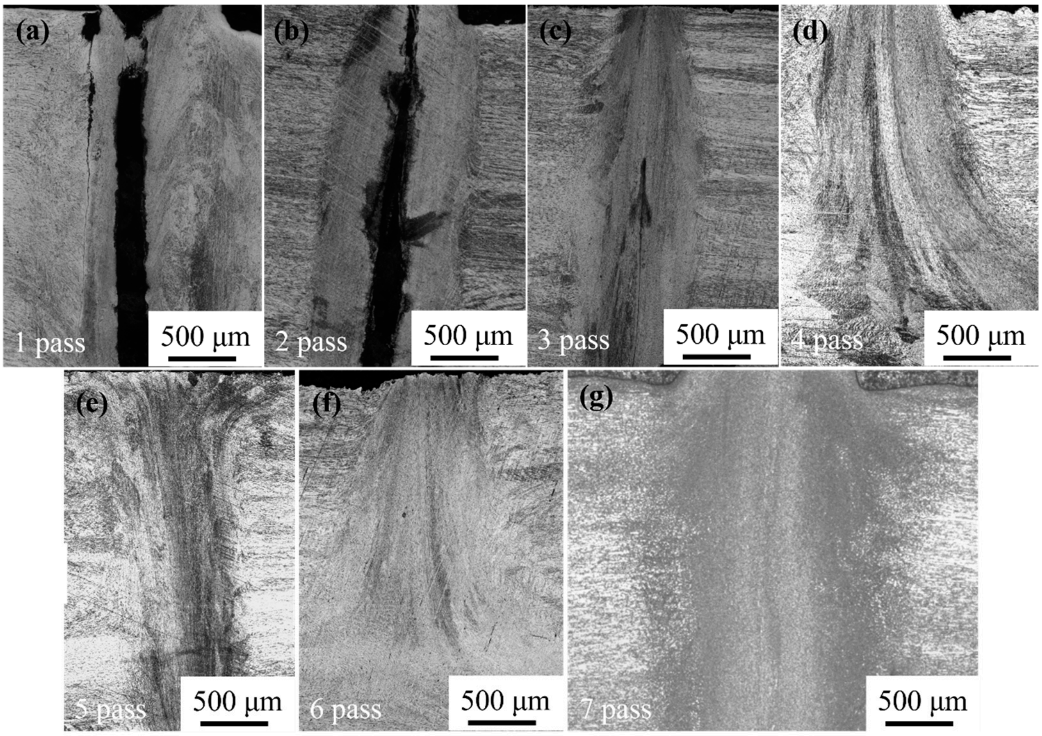

Figure S7. (**a**–**g**) Optical micrographs of Cu-Mg wire joints that have undergone 1–7 upsetting cold-welding processes, respectively.
